# Mélanocytome méningé: évolution agressive d’une tumeur bénigne: à propos de 2 cas

**DOI:** 10.11604/pamj.2018.29.211.10680

**Published:** 2018-04-11

**Authors:** Mohamed Khoulali, Mohammed Yasssine Haouas, Jihad Mortada, Robin Srour

**Affiliations:** 1 Université Mohammed Premier, Faculté de Médecine et de Pharmacie, CHU Mohamed VI, Oujda, Maroc; 2 Université Hassan II, CHU Ibn Rochd, Casablanca, Maroc; 3 Hôpital Louis Pasteur, Colmar, France; 4 Hôpital Louis Pasteur, Colmar, France

**Keywords:** Mélanocytome méningé, transformation maligne, métastase leptoméningée, Meningeal melanocytoma, malignant transformation, leptomeningeal metastasis

## Abstract

Les mélanocytomes méningés sont des tumeurs pigmentées rares qui affectent le système nerveux central et se développent dans les leptoméninges cérébrospinales. Nous présentons deux cas de mélanocytomes méningés montrant une très grande diversité évolutive: une très longue évolution locale d'une lésion méningée cérébrale, avec transformation maligne ayant entrainé le décès après 32 ans pour le premier malade, et la localisation intra-médullaire ectopique à diffusion méningée massive très rapide, pour la deuxième malade. Ces deux cas montrent le profil évolutif incertain des mélanocytomes méningés et ces lésions peuvent devenir agressives dont le pronostic est sombre malgré des thérapeutiques intensives.

## Abstract (en)

Meningeal melanocytomas are rare pigmented tumors affecting the central nervous system and developing in the cerebrospinal leptomeninges. We report two cases of meningeal melanocytomas showing very marked disparity in their evolution: a very long-term development of meningocerebral lesion, with malignant transformation resulting in the death of the first patient after 32 years and intramedullary ectopic location with very fast massive meningeal diffusion in the second patient. These two cases show the uncertain evolutive profile of meningeal melanocytomas. These lesions may become aggressive with poor prognosis despite an intensive therapeutic strategy.

## Introduction

Les mélanocytomes méningés sont des tumeurs très rares du système nerveux central. Ils se distinguent par des caractères évolutifs particuliers, et très incertains. Nous rapportons deux observations; un homme de 43 ans qui présente un mélanocytome méningé retro-orbitaire droit siégeant au niveau du cavum de Meckel ayant évolué vers la malignité et la dissémination méningée massive; une femme âgée de 51 ans qui présente un mélanocytome méningé ectopique, médullaire, de découverte fortuite, et qui a pris le même caractère évolutif, aboutissant aux décès des deux patients dans les mois qui ont suivi leurs disséminations méningées.

## Patient et observation

### Cas 1

**Figure 1:**
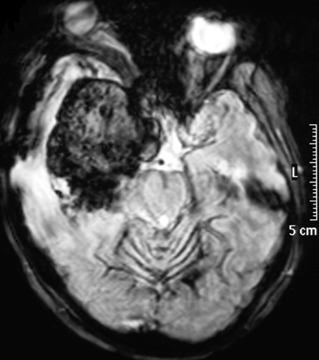
IRM cérébrale: coupe axiale séquence T2, montrant une lésion hypointense au niveau du cavum de Meckel s'étendant vers l'orbite

**Figure 2:**
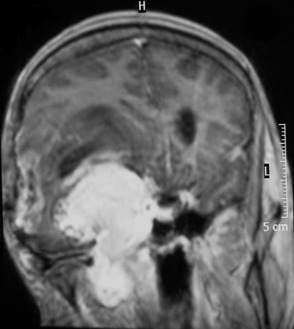
IRM cérébrale: en coupe coronale séquence T1 après injection du gadolinium, montrant une lésion au niveau du cavum de Meckel s'étendant vers la fosse infra-temporale et les muscles ptérygoïdiens; la lésion se rehausse de façon intense et homogène après injection du Gadolinium

**Figure 3:**
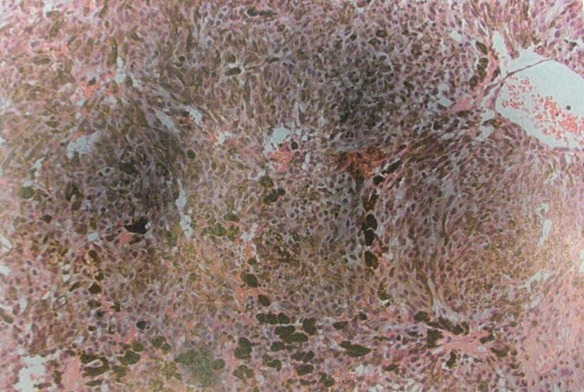
Aspect histologique de la tumeur visualisation de cellules hétérogènes et irrégulières avec composante fusiformes, présences de mitoses et surcharge mélanique visible en brun

Mr E.M, âgé de 42 ans, il présentait depuis sa naissance, une asymétrie des fentes palpébrales. A l'âge de 17 ans, il a été opéré d'une tumeur méningée sphéno-orbitaire droite, qui s'est manifestée par des douleurs retro-orbitaire droite. Le diagnostic histologique était celui d'une mélanomatose méningée, pire que le diagnostic soit resté imprécis. L'évolution est restée stable pendant 25 ans. Les documents radiologiques qui ont été effectués durant cette période ont été détruits dans un incendie. En raison d'une exophtalmie avec ophtalmoplégie droite et baisse de l'acuité visuelle rapidement progressive sur 3 mois, une nouvelle IRM cérébrale, comprenant des séquences pondérées T1 et T2, puis T1 après injection de gadolinium dans les trois plans a été réalisée. Elle montrait une lésion extra-axiale développée au niveau du sinus caverneux droit, s'étendant vers l'orbite avec une extension vers la fosse infra-temporale et les muscles ptérygoïdiens. Cette lésion apparaissait bien limitée, sans signes d'envahissement cérébral et présentait un signal globalement hyperintense en T1 et hypointense en T2. La lésion prenait le contraste de manière homogène et intense (Figure 1, Figure 2). L'intervention chirurgicale a été réalisée par un abord ptérional, macroscopiquement, il s'agissait d'une tumeur noirâtre molle et gélatineuse très hémorragique, dont l'exérèse était subtotale. En microscopie, la tumeur était constituée de cellules fusiformes avec des noyaux ovalaires discrètement irréguliers et un cytoplasme abondant apparaissant rempli de pigments marqués par la coloration de Fontana (coloration de la mélanine). Les mitoses étaient présentes en nombre supérieur à trois pour dix champs à fort grossissement. Il n'y avait pas de nécrose. En immunohistochimie, les cellules étaient positives pour HMB45. L'index mitotique évalué avec l'anticorps anti-Ki 67 était de 20% (Figure 3, ). Une radiothérapie adjuvante était proposée mais l'évolution est marquée, un mois après l'intervention par l'apparition d'une méningite d'allure bactérienne traitée par antibiothérapie adaptée. Deux semaines après, le patient présentait une aggravation neurologique brutale. Le bilan neuroradiologique montrait une énorme récidive tumorale avec diffusion leptoméningée massive associée a une hémorragie intra parenchymateuse et intra ventriculaire et l'état neurologique restait précaire jusqu'au décès, deux mois plus tard soit moins d'un an après la réapparition des signes cliniques, et vingt-cinq ans après la première intervention.

### Cas 2

**Figure 4:**
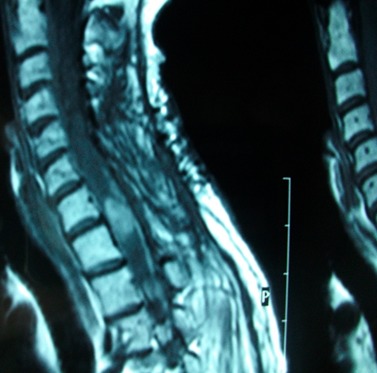
IRM cervicale en coupe sagittale séquence T1, montrant une lésion hyperintense intramédullaire siégeant en regard de C7-T1

**Figure 5:**
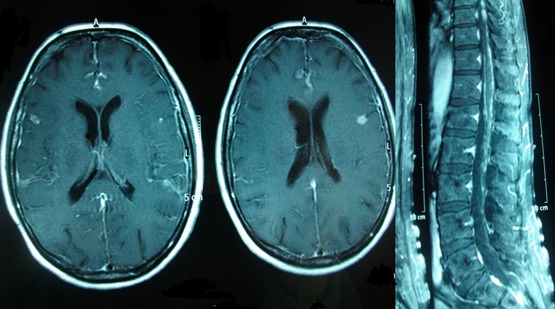
IRM cérébro-spinale après injection du gadolinium réalisée après 4 ans d'évolution, montrant une prise de contraste leptoméningée diffuse sur tout l'axe cérébro-spinale

Mme A.W âgée de 51 ans sans antécédents pathologique notable avait bénéficié d'une IRM cervicale dans les suites d'un accident de la voie publique responsable d'un traumatisme cranio cervical avec des paresthésies des 04 membres spontanément dégressives. L'IRM médullaire cervicale avait mis en évidence une lésion hyperintense ayant évoqué seulement le diagnostic d'une contusion médullaire dans ce contexte post traumatique (Figure 4). L'absence d'évolution radiologique et les discordances radio cliniques ont fait proposer, secondairement, le diagnostic de cavernome intra médullaire cervicale, et une surveillance radio clinique annuelle était proposée. Elle est restée asymptomatique pendant 4 ans jusqu'à l'apparition de céphalées cervico-occipitale avec un flou visuel et la découverte d'un œdème papillaire bilatérale à l'examen du fond d'œil. Le bilan neuroradiologique montrait cette fois ci une lésion déjà connue très discrètement augmentée de volume et surtout la dissémination méningée sur tout l'axe cérébrospinal (Figure 5). L'intervention a porté sur la lésion médullaire cervicale où une biopsie a été réalisée. En microscopie la tumeur était constituée par des cellules ovales avec des noyaux irréguliers et cytoplasme riche en mélanine sans atypie cytonucléaire et à faible indice prolifératif. L'immuno-marquage HMB 45 a confirmé la présence de mélanocytes. Le bilan d'extension postopératoire réalisé (consultation dermatologique, TDM thoraco-abdominopelvien, scintigraphie osseuse et PET-scanner) a permis de retenir le diagnostic d'un mélanocytome méningé médullaire primitif compliqué d'une mélanocytose méningée diffuse secondaire, responsable de l'hypertension intracrânien. Apres avis de plusieurs Réunion de concertation pluridisciplinaire, une irradiation cérébrospinale associée à une chimiothérapie par Temozolomide ont permis une réponse initiale satisfaisante, avec diminution des céphalées. Néanmoins, un syndrome confusionnel avec majoration des céphalées, et coma réapparut de façon subaigüe. Deux mois après le début de la chimiothérapie, celle-ci ne put être poursuivie en raison de l'aggravation de l'état général de la patiente. La patiente décéda en moins de trois mois.

## Discussion

Les tumeurs mélanocytaires sont des lésions rares du système nerveux central, représentant 0,1% des tumeurs cérébrales. Elles sont développées à partir des mélanocytes des leptoméninges dérivant de la crête neurale. L'originalité des cas rapportés repose sur le caractère évolutif imprévisible du mélanocytome. Les mélanocytomes sont en général des tumeurs bénignes. Bien que exceptionnel, une transformation maligne et un envahissement leptoméningé diffus sont possibles [1-3]. Ils surviennent à tout âge avec un pic lors de la cinquième décade, avec une discrète prédominance féminine [4]. La plupart des mélanocytomes se développe dans le compartiment intradural extramédullaire au niveau du rachis cervical et thoracique [5]. De façon moins fréquente, ils surviennent dans les leptoméninges supra-tentorielles, dans la fosse postérieure ou le cavum de Meckel [6]. La symptomatologie neurologique dépend de la localisation tumorale. Le scanner montre un aspect hyperdense, se rehaussant après injection du produit de contraste. Les calcifications et les réactions osseuses sont exceptionnelles. L'IRM est caractéristique du fait des propriétés paramagnétiques de la mélanine: signal hyperintense en T1 et hypointense en T2, et une prise de contraste intense et homogène. Cependant, cet aspect est inconstant et dépendrait de la richesse tumorale en mélanine [7]. La présence d'une prise de contraste leptoméninge sur l'axe cérébrospinal indique la diffusion tumorale. Macroscopiquement, c'est une tumeur noirâtre ou brune à pigmentation marquée par la coloration de Fontana. Finalement, c'est l'examen histologique qui permet d'affirmer la nature mélanocytaire de la lésion. Il permet également de distinguer les lésions primitives de bas grade et de grade intermédiaire, des lésions secondaires qui sont toujours de haut grade. L'examen histologique peut être pris en défaut pour les lésions de haut grade dont le caractère primitif ou secondaire ne sera souvent supposé qu'au vu d'un bilan d'extension exhaustif [8]. La présence d'une invasion du système nerveux central, d'une cellularité accrue, d'un pléiomorphisme et d'une activité mitotique accrue doit être signalée dans le compte-rendu histologique pour permettre de classer ces tumeurs au sein du spectre des tumeurs mélanocytaires du système nerveux central allant du mélanocytome au mélanome [4]. La plupart des mélanocytes expriment les marqueurs mélaniques (HMB 45, Melan-A) ainsi que la protéine S100. Le marquage avec la vimentine est plus variable. L'index de prolifération évalué à l'aide du KI67 n'excède pas 1 à 2%. Récemment, Zembowicz et al. ont montré qu'il existait une perte d'expression de la sous-unité 1 alpha de la protéine kinase A, codée par le gène PRKAR1A situé en 17q22-24 dans les mélanocytomes épithélïoides pigmentés contrairement au mélanome ou aux autres lésions mélanocytaires pouvant mimer un mélanocytome. Ainsi l'immuno-histochimie avec l'anticorps R1alpha est une aide au diagnostic [9]. Ce marquage n'a pas pu être réalisé dans nos observations.

Le diagnostic différentiel des tumeurs mélanocytaires leptoméningées localisées se pose avec les neurinomes et les méningiomes, alors que les métastases d'un mélanome posent le problème de diagnostic différentiel avec les formes diffuses. Le traitement des mélanocytomes du système nerveux central repose sur une exérèse chirurgicale complète si possible, constituant le meilleur facteur pronostic. Une radiothérapie est discutée en cas d'exérèse incomplète après reprise chirurgicale d'un mélanocytome de bas grade mais elle est également recommandée pour les patients porteurs d'un mélanocytome de haut grade, focalisée sur la lésion à une dose de 50-52,5 GRY, 1,8 a 2 GY par fraction est significativement efficace. La radiochirurgie Gamma-Kniffe a été utilisée avec succès pour des tumeurs de petites tailles. La chimiothérapie est indiquée pour les lésions de haut grade. Plusieurs molécules ont été utilisées sans preuve d'efficacité. L'évolution clinique des mélanocytomes est parfois marquée par des récidives locales. La transformation maligne et la diffusion méningée des mélanocytomes sont exceptionnelles [1-3]. Nos deux patients montrent la très grande diversité évolutive des mélanocytomes méningée: très longue évolution locale d'une lésion méningée cérébrale, avec transformation maligne ayant entrainé le décès après 32 ans pour le premier malade, et la localisation intra-médullaire ectopique à diffusion méningée massive très rapide, pour la deuxième malade.

## Conclusion

Bien que bénigne, l'évolution des tumeurs mélanocytaires méningées primitives vers la transformation maligne et la diffusion leptoméningée est imprévisible, d'où l'importance de poser un diagnostic précoce et mise en place d'une thérapeutique adaptée; bien que non protocolisée, basée sur l'exérèse chirurgicale complète, le meilleur facteur pronostic.

## Conflits d’intérêts

Les auteurs ne déclarent aucun conflit d'intérêts.
